# Supplementary material for: The Stairway to Antibiotic Heaven: A Scaffolded Video Series on Empiric Antibiotic Selection for Fourth-Year Medical Students
Source: MedEdPORTAL. 2020 Nov 30;16:11036. doi: 10.15766/mep_2374-8265.11036 (PMC7703485; doi:10.15766/mep_2374-8265.11036)
Supplement: Supplementary file 1 — Video 1-Introduction.mp4Video 2-Amoxicillin.mp4Video 3-Ceftriaxone.mp4Video 4-Vancomycin and Azithromycin.mp4Video 5-Piperacillin-Tazobactam and Ampicillin-Sulbactam.mp4Video 6-Cefepime.mp4Video 7-Aminoglycosides.mp4Video 8-Carbapenems.mp4Embedded Questions.docxPre- and Posttest Question Bank.docxPostvideo Survey.docx [file mep_2374-8265.11036-s001.zip › _Educational_Summary_Report_11036.pdf]

MedEdPORTAL® | The AAMC Journal of Teaching and Learning Resources

Original Publication

Open Access

The Stairway to Antibiotic Heaven: A Scaffolded Video Series on Empiric Antibiotic Selection for Fourth-Year Medical Students

Jeffrey Larnard, MD*, Jason Zucker, MD, Rachel Gordon, MD, MPH

*Corresponding author: jclarnar@gmail.com

Abstract

Introduction: Inappropriate antibiotic use and spread of resistance is a well-known problem, and medical students have indicated they want additional education on appropriate use of antimicrobials. We introduced a series of short whiteboard animation videos on empiric antibiotic selection as a supplemental resource for fourth-year medical students during a transition to residency course. **Methods:** A total of eight whiteboard animation videos on empiric antibiotic selection were created using Camtasia. The video series started with the narrowest spectrum antibiotic discussed and progressed up an antibiotic ladder to broader spectrum antibiotics. Questions were embedded in each video. Students were offered a pretest prior to viewing the video series as well as a posttest after completing the video series. After each individual video, students were offered a postvideo survey with Likert-scaled questions evaluating student perceptions of the video. All tests and surveys were anonymous. Scores of pre- and posttests were compared with unpaired *t* tests. **Results:** We received 37 pretests and 14 posttests. The average score on the pretest was 66% compared with 93% on the posttest ($p < .0001$; 95% CI 16.78, 37.93). Seventy-two postvideo surveys were completed across all videos. Of student responses, 100% either agreed or strongly agreed that the evaluated module was an effective way to learn the material. **Discussion:** Our results suggested that this scaffolded, interactive video animation series on antibiotic spectrum and selection was an effective learning activity.

Keywords
Antibiotic Stewardship, Animation, Antibiotics, Pharmacology & Toxicology, Multimedia, Online/Distance Learning

Educational Objectives

By the end of this activity, learners will be able to:

1. Compare and contrast the spectrum of activities of different antibiotics.
2. Explain why certain antibiotics are used as empiric antibiotic therapy in different clinical scenarios.
3. Select appropriate empiric antibiotic therapy in various clinical scenarios.

Introduction

Inappropriate antibiotic use and spread of resistance is a well known global problem, but despite this, the decision to start antibiotics is primarily left to nonspecialists.[1] A 2017 study of internal medicine residents found that residents perceived they were often responsible for initial empiric antibiotic prescriptions.[2] Despite the role that resident physicians have in initiating antibiotics, in a study of medical students from three different US medical schools, 90% of respondents indicated that they would like more education on appropriate use of antimicrobials. Moreover, respondents suggested that additional antimicrobial education should occur in the clinical years.[3] The Infectious Diseases Society of America and The Society of Healthcare Epidemiology of America also suggested that antimicrobial stewardship principles should be integrated into curricula for medical students when implementing an antibiotic stewardship program and advised against relying solely on didactic methods.[4]

Citation:
Larnard J, Zucker J, Gordon R. The stairway to antibiotic heaven: a scaffolded video series on empiric antibiotic selection for fourth-year medical students. *MedEdPORTAL*. 2020;16:11036.
https://doi.org/10.15766/mep_2374-8265.11036

Using videos to deliver educational content may offer advantages over verbal communication including the ability to present more information in a given amount of space and time and to simplify complex content. Videos also may be more effective at gaining audience attention.[5] Whiteboard animations are videos that show the process of drawing a finished picture, generally on something resembling a traditional whiteboard. They are able to place the viewer in the narrator role as images are constructed with the goal of helping the learner also mentally construct the concept.[6]

Copyright © 2020 Larnard et al. This is an open-access publication distributed under the terms of the Creative Commons Attribution license.

Whiteboard animations are also able to utilize colors and shapes as a way to affect the learner's emotions and foster learning.[7]

Though there have been publications in *MedEdPORTAL* that used video as an educational medium for medical students,[8,9] to our knowledge there were none focusing on appropriate antimicrobial use. Overall, despite the increasing awareness of the importance of appropriate antibiotic use, there has been a relative paucity of resources published for medical students. One recent study in *MedEdPORTAL* used an engaged learning module to teach medical students foundational concepts in antimicrobial stewardship,[10] but did not focus on the spectrum of activity of antimicrobials, which is of utmost practical importance.

We created a whiteboard animation video series on empiric antibiotic selection and escalation and made it available as an optional, supplemental resource to fourth-year medical students at Columbia University Vagelos College of Physicians and Surgeons (CU VP&S). The video series was offered as part of the blended, transition to residency course Ready 4 Residency. This course combined in-person didactic instruction with online and simulation components with the goal of preparing fourth-year medical students for intern year. We aimed to see if the video series could increase knowledge of antibiotic coverage as well as investigate student's perceptions of the whiteboard animation video series.

Methods

We created a total of eight whiteboard animation style videos (Appendices A-H). The video series included: (1) an introductory video, followed by (2) videos on amoxicillin, (3) ceftriaxone, (4) vancomycin and azithromycin, (5) piperacillin-tazobactam and ampicillin-sulbactam, (6) cefepime, (7) aminoglycosides, and (8) carbapenems. First, we made scripts for each of the videos based on what was originally a popular chalk talk given by one of the authors to medical students and residents. We used the iPad application Show Me as the whiteboard while we accomplished the screen capture, editing, and layering of vocal narration using Camtasia. All videos were less than 7 minutes long and most were shorter than 6 minutes. We designed the videos to be as concise as possible in an effort to retain the maximum amount of learners throughout the entire video.

The videos used a pediatric pneumonia case to scaffold the learning using the antibiotic ladder seen in Figure 1. The students

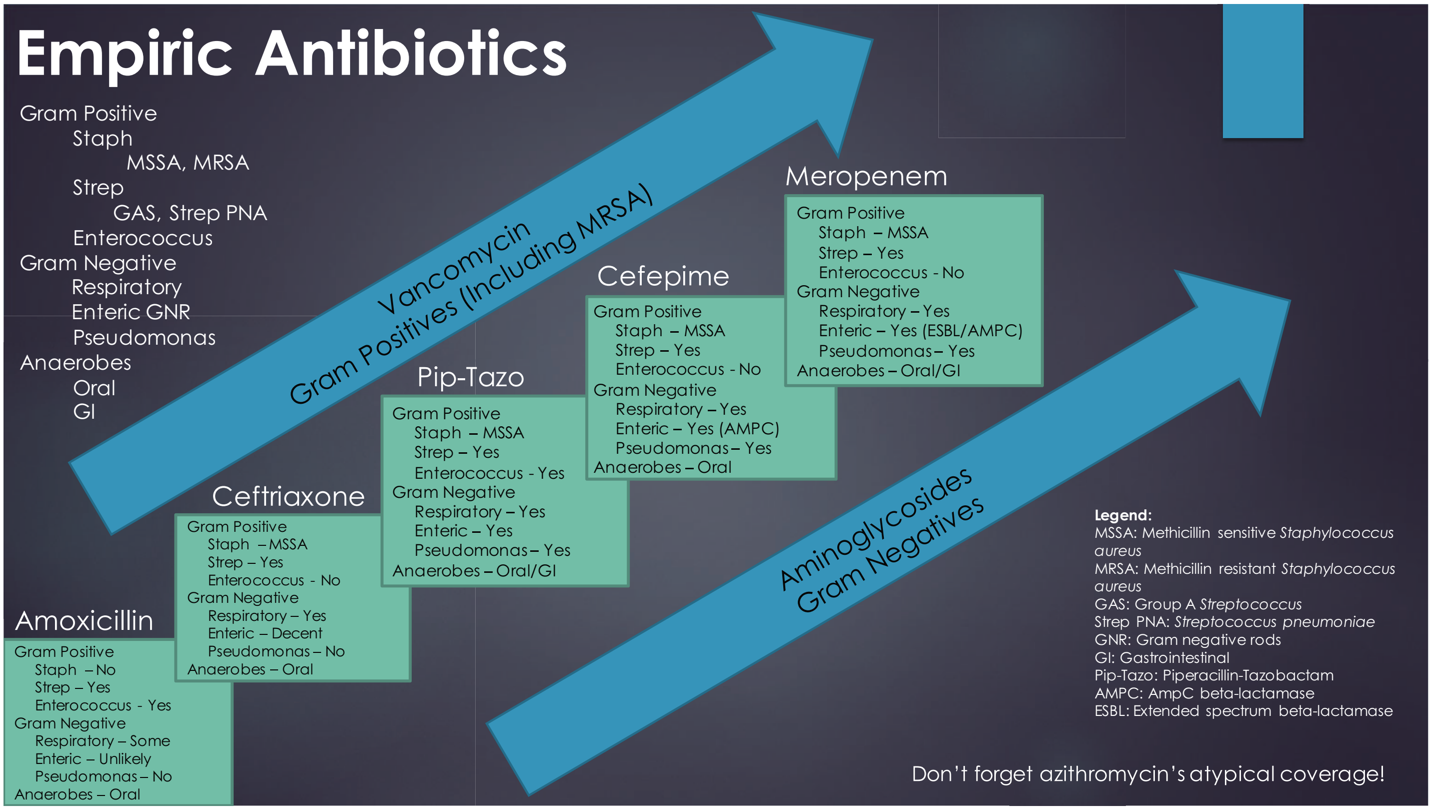

Figure 1. Antibiotic ladder used in video series.

Copyright © 2020 Larnard et al. This is an open-access publication distributed under the terms of the Creative Commons Attribution license.

started learning the narrowest spectrum antibiotic in the series and progressed up the ladder in subsequent videos to the broadest spectrum antibiotic in the series. The ladder itself was featured towards the end of each video in the hope of not only reviewing the coverage of the featured antibiotic, but also to provide spaced learning opportunities by revisiting the coverage of the previous antibiotic. The videos also included multiple choice questions interspersed throughout the videos that included feedback for correct and incorrect answer choices. We utilized both spaced learning and practice questions as they have been shown to be more effective ways of learning information compared to other common methods of study.[11] Each video also featured a 2x2 table for each antibiotic which highlighted its Gram positive, Gram negative, atypical, and anaerobic coverage.

We included all videos in the appendix as MP4 files (Appendices A-H) though it should be noted that in this format they were not able to have questions embedded. The questions that were embedded in the Camtasia videos were included separately in Appendix I. Ideally, students would encounter the questions within the videos themselves, but educators have the option to distribute Appendix I to students prior to watching the videos so students can answer the questions at the end of each video as they progress through the series.

The videos were implemented as an optional, supplemental resource for fourth-year medical students at CU VP&S during Ready 4 Residency in April 2019. Approval was obtained by the Columbia University Institutional Review Board (IRB-AAAS3444). The video series was made as a review and it was presumed that students were at least somewhat familiar with the antibiotics discussed from earlier courses or clinical rotations. A total of 72 students were enrolled in Ready 4 Residency during the period in which the videos were offered.

Students were offered an untimed, nonmandatory 8-question pretest before starting the video series and an untimed, nonmandatory 8-question posttest after completing the video series. There were four questions written for each of the eight videos, thus the question bank consisted of 32 total questions (Appendix J). Qualtrics was used to block randomize questions from the common question bank so each test would contain one of the four available questions for each video (eight total questions) and so the pre- and posttests may contain different questions. Questions were generated by the primary author and reviewed by the other authors who are both faculty in the Division of Infectious Disease. Pre- and posttests were completely anonymous and were compared using unpaired *t* tests.

Students were also offered anonymous postvideo surveys after each video that had several Likert-scaled statements (response options were *strongly disagree*, *disagree*, *neutral*, *agree*, and *strongly agree*) as well as an option to free text feedback (Appendix K). The first set of Likert-scaled statements focused on student perception of the video, while the second set of Likert-scaled statements addressed the learning objectives of the video series. Both sets of statements can be found in the Table.

Results

Thirty-seven pretests and 14 posttests were completed by fourth-year medical students during the Ready 4 Residency course in April 2019. Out of the 72 students enrolled in Ready 4 Residency, 51% took the pretest and 19% took the posttest, with the assumption that students did not take either more than once. The average score on the pretest was 66% compared with 93% on the posttest ($p < .0001$; 95% CI 16.78, 37.93), as shown in Figure 2.

Seventy-two postmodule surveys were also completed by fourth-year medical students during Ready 4 Residency across the eight videos. We do not know the response rate as some of the surveys were likely completed by the same student for different videos. Overall, the majority of responses indicated that students

Table. Breakdown of Responses to Statements Regarding Student Perception of the Video Series and the Learning Objectives ($n = 72$)

Statement	Strongly Disagree (%)	Disagree (%)	Neutral (%)	Agree (%)	Strongly Agree (%)
This video module was an effective way to learn about antibiotic coverage.	0	0	0	51	49
The length of this video module was appropriate.	0	0	1	38	61
This video module had an appropriate level of difficulty.	0	0	1	46	53
I enjoyed this video module.	0	0	1	52	47
The questions within the video module were a helpful way to reinforce the material.	0	0	1	35	64
After watching this module, I am more likely to remember the spectrum of activity of the presented antibiotics.	0	1	8	46	45
After watching this module, I better understand why the presented antibiotics are selected for empiric coverage.	0	1	8	38	53
After watching this module, I feel more comfortable using the presented antibiotics in the appropriate clinical setting.	0	1	9	50	40

Copyright © 2020 Larnard et al. This is an open-access publication distributed under the terms of the Creative Commons Attribution license.

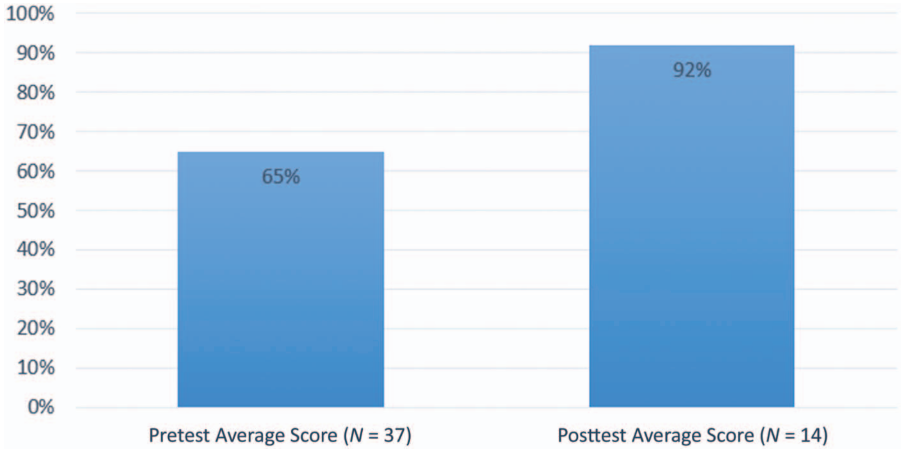

Figure 2. Comparison of the average scores on pretests versus posttests taken by fourth-year medical students. The difference between the pre- and posttests for fourth-year medical students was significant ($p < .0001$; 95% CI 16.78, 37.93).

agreed that the modules were an effective way to learn about antibiotic coverage and that the videos had an appropriate length and difficulty as seen in the Table. Responses also noted that the questions within the video were a helpful way to reinforce the material. In the second set of Likert-scaled statements, most students responded that they either *agree* or *strongly agree* that after watching the video they were more likely to remember the spectrum of activity of the presented antibiotics (46% and 45%, respectively) and felt more comfortable using the presented antibiotics (50% and 40%, respectively; Table).

There were also 11 total comments received in the free text portion of the survey. Of comments, 10 out of the 11 were positive and the other respondent commented that they wanted additional learning on resistant organisms. Students liked that the videos contextualized empiric antibiotic coverage and escalation with one representative comment stating, “I had picked up bits and pieces of this on my rotations and just by watching my residents/attendings mentally run through this ‘staircase,’ but no one had laid it out for me like this.” Students also mentioned the questions embedded in the videos with another representative comment stating, “I appreciate the questions inserted near the end to test our understanding and comprehension of the information.”

Discussion

Our results suggested that this scaffolded and interactive animated video series was an effective way to teach empiric antibiotic selection and escalation for medical students during their clinical years. The improvement between the pre- and posttests was significant and postmodule survey responses also indicated that the videos were an effective way to learn the material. The vast majority of responses also indicated that the videos were an appropriate length and difficulty and found the embedded questions to be helpful. Qualitative feedback for the video series was also positive. Since the conclusion of the study, we have maintained the video series as an optional, supplemental resource during Ready 4 Residency and also offered the video series to preclinical medical students, clinical medical students, and interns.

Users of this video series will be able to upload the series to a learning management system. Educators can distribute the questions in Appendix I to students prior to viewing the videos with the goal of having students answer the questions as they progress through the series. As we noted in the Methods section, we were unfortunately not able to make the videos with embedded questions available for upload because the ability to embed questions relies on a specific Camtasia web player. We hope that having the questions available in some form may still allow students to test themselves as well as help them identify key information in each of the videos.

A major limitation of our study was the small sample size and that there were more pretests than posttests completed. Our results should be viewed with the knowledge that, since the tests were completely anonymous, it was impossible to tell which of the students who took the pretest ended up completing the video series. It is possible that the students who performed worse on the pretest were less likely to complete the video series, and students who completed the video series and subsequent posttest may have self-selected the strongest students. Going forward, keeping the tests anonymous but adding identifiers to the pre- and posttests could help better compare the results.

Copyright © 2020 Larnard et al. This is an open-access publication distributed under the terms of the Creative Commons Attribution license.

We also acknowledge that more feedback could be elicited from the participants of the study to continue to improve our video series. We only received 11 comments in the free text portion of the postmodule survey, and it was beneficial when participants shared specific feedback. One respondent indicated they wanted additional teaching on resistant organisms, which helped us start planning a new video focusing on novel antibiotics and how these agents can cover multidrug-resistant bacteria. It will be valuable to continue to seek out additional feedback from trainees with more specific free text questions or focus groups.

Future directions include whether the video series can be an effective learning resource for other trainees and providers including preclinical medical students, interns, residents, and advanced practitioners. Our videos focused on the prescriber role, but as antibiotic prescribing is often a multidisciplinary collaboration, future videos should include other roles in the care team. We are also interested in seeing whether knowledge gains eventually translate into clinical practice. Our study focused on knowledge acquisition and perception of the educational material, but it would be relevant in the future to see if our video series could actually influence practice. Further studies could additionally explore whether animated and interactive videos can be an effective way to deliver other educational content in undergraduate medical education, knowing that the time and effort to produce these videos is a potential barrier to widespread use.

Appendices

A. Video 1—Introduction.mp4
B. Video 2—Amoxicillin.mp4
C. Video 3—Ceftriaxone.mp4
D. Video 4—Vancomycin and Azithromycin.mp4
E. Video 5—Piperacillin-Tazobactam and Ampicillin-Sulbactam.mp4
F. Video 6—Cefepime.mp4
G. Video 7—Aminoglycosides.mp4
H. Video 8—Carbapenems.mp4
I. Embedded Questions.docx
J. Pre- and Posttest Question Bank.docx
K. Postvideo Survey.docx

All appendices are peer reviewed as integral parts of the Original Publication.

Jeffrey Larnard, MD: Resident, Department of Medicine, Columbia University Irving Medical Center

Jason Zucker, MD: Instructor, Department of Medicine, Columbia University Irving Medical Center

Rachel Gordon, MD, MPH: Associate Professor, Department of Medicine and Epidemiology, Columbia University Irving Medical Center

Disclosures
None to report.

Funding/Support
None to report.

Prior Presentations
Larnard J, Zucker J, Gordon R. The stairway to antibiotic heaven: evaluating a scaffolded video series on empiric antibiotic selection. Poster presented at: IDWeek; October 5, 2019; Washington, DC.

Ethical Approval
The Columbia University Institutional Review Board approved this study.

References

1. Charani E, Cooke J, Holmes A. Antibiotic stewardship programmes—what's missing? *J Antimicrob Chemother*. 2010;65(11):2275-2277. https://doi.org/10.1093/jac/dkq357
2. Laake AM, Bernabe G, Peterson J, Liappis AP. Internal medicine resident perspectives regarding broad-spectrum antibiotic usage. *Open Forum Infect Dis*. 2017;4(2):ofx060. https://doi.org/10.1093/ofid/ofx060
3. Abbo LM, Cosgrove SE, Pottinger PS, et al. Medical students' perceptions and knowledge about antimicrobial stewardship: how are we educating our future prescribers? *Clin Infect Dis*. 2013;57(5):631-638. https://doi.org/10.1093/cid/cit370
4. Barlam TF, Cosgrove SE, Abbo LM, et al. Implementing an antibiotic stewardship program: guidelines by the Infectious Diseases Society of America and the Society for Healthcare Epidemiology of America. *Clin Infect Dis*. 2016;62(10):e51-e77. https://doi.org/10.1093/cid/ciw118
5. Hurtubise L, Martin B, Gilliland A, Mahan J. To play or not to play: leveraging video in medical education. *J Grad Med Educ*. 2013;5(1):13-18. https://doi.org/10.4300/JGME-05-01-32
6. Türkay S.. The effects of whiteboard animations on retention and subjective experiences when learning advanced physics topics. *Comput Educ*. 2016;98:102-114. https://doi.org/10.1016/j.compedu.2016.03.004
7. Plass JL, Heidig S, Hayward EO, Homer BD, Um E. Emotional design in multimedia learning: effects of shape and color on affect and learning. *Learn Instr*. 2014;29:128-140. https://doi.org/10.1016/j.learninstruc.2013.02.006
8. Dominguez M, DiCapua D, Leydon G, et al. A neurology clerkship curriculum using video-based lectures and just-in-time teaching

Copyright © 2020 Larnard et al. This is an open-access publication distributed under the terms of the Creative Commons Attribution license.

(JiTT). *MedEdPORTAL*. 2018;14:10691. https://doi.org/10.15766/mep_2374-8265.10691

9. Rana J, Mostaghimi A. Introduction to skin cancer: a video module. *MedEdPORTAL*. 2016;12:10431. https://doi.org/10.15766/mep_2374-8265.10431

10. Hsu JL. Building an antibiotic stewardship program: an interactive teaching module for medical students. *MedEdPORTAL*. 2018; 14:10726. https://doi.org/10.15766/mep_2374-8265.10726

11. Dunlosky J, Rawson KA, Marsh EJ, Nathan MJ, Willingham DT. Improving student's learning with effective learning techniques: promising directions from cognitive and educational psychology. *Psychol Sci Public Interest*. 2013;14(1):4-58. https://doi.org/10.1177/1529100612453266

Received: February 9, 2020
Accepted: July 30, 2020
Published: November 30, 2020

Copyright © 2020 Larnard et al. This is an open-access publication distributed under the terms of the Creative Commons Attribution license.
