# Supplementary material for: The Stairway to Antibiotic Heaven: A Scaffolded Video Series on Empiric Antibiotic Selection for Fourth-Year Medical Students
Source: MedEdPORTAL. 2020 Nov 30;16:11036. doi: 10.15766/mep_2374-8265.11036 (PMC7703485; doi:10.15766/mep_2374-8265.11036)
Supplement: Supplementary file 1 — Video 1-Introduction.mp4Video 2-Amoxicillin.mp4Video 3-Ceftriaxone.mp4Video 4-Vancomycin and Azithromycin.mp4Video 5-Piperacillin-Tazobactam and Ampicillin-Sulbactam.mp4Video 6-Cefepime.mp4Video 7-Aminoglycosides.mp4Video 8-Carbapenems.mp4Embedded Questions.docxPre- and Posttest Question Bank.docxPostvideo Survey.docx [file mep_2374-8265.11036-s001.zip › I. Embedded Questions.docx]

Embedded Questions:

**Video 1 - Introduction:**

Question 1:

You are treating a patient on the general medicine service with a community acquired pneumonia. The Gram stain for the respiratory culture results as Gram negative coccobacilli. Which of the following is most likely to be the potential pathogen?

**A. *Haemophilus influenzae***

B. *Legionella pneumophila*

C. *Listeria monocytogenes*

D. *Mycoplasma pneumoniae*

E. *Staphylococcus aureus*

Question 2:

You are treating an outpatient pediatric patient for community acquired pneumonia with a beta-lactam antibiotic. Which of the following will not be covered by beta-lactam antibiotics?

A. *Enterococcus faecalis*

B. *Haemophilus influenzae*

C. *Moraxella catarrhalis*

**D. *Mycoplasma pneumoniae***

E. *Streptococcus pyogenes*

Question 3:

You are treating a young woman for a urinary tract infection and are most concerned about enteric Gram negative rods. Which of the following organisms is most likely to be the culprit?

A. *Enterococcus faecalis*

B. *Listeria monocytogenes*

C. *Moraxella catarrhalis*

**D. *Escherichia coli***

E. *Streptococcus pyogenes*

Question 4:

CHALLENGE QUESTION: What is the antibiotic of choice to treat outpatient pediatric community acquired pneumonia?

**A. Amoxicillin**

B. Ampicillin

C. Ceftriaxone

D. Piperacillin-tazobactam

E. Meropenem

**Video 2 – Amoxicillin:**

Question 1:

You prescribe CeCe amoxicillin because you are concerned about the most common bacteria implicated in pediatric (and adult) CAP. What is the most likely organism?

A. *Haemophilus Influenzae*

B. *Moraxella catarrhalis*

C. *Mycoplasma pneumoniae*

D. *Staphylococcus aureus*

**E. *Streptococcus pneumoniae***

Question 2:

You are treating a clinic patient for a post-influenza bacterial pneumonia. You are understandably concerned about *S aureus* and decide against using amoxicillin. The most common reason why *S aureus* is resistant to amoxicillin is that they...

A. change their penicillin binding proteins.

B. do not have peptidoglycan cell walls.

C. have thickened cell membranes.

**D. produce beta-lactamases.**

E. pump it out (with efflux pumps).

Question 3:

You are treating an outpatient pediatric patient with amoxicillin for presumed pneumonia. Which of the following organisms is most likely to be covered by amoxicillin?

A. *Chlamydophila pneumoniae*

**B. *Haemophilus influenzae***

C. *Legionella pneumophila*

D. *Moraxella catarrhalis*

E. *Staphylococcus aureus*

**Video 3 - Ceftriaxone:**

Question 1:

Which of the following beta-lactam antibiotic would be the most appropriate in this scenario (treating inpatient community acquired pneumonia)?

A. Ampicillin

B. Ampicillin-Sulbactam

**C. Ceftriaxone**

D. Meropenem

E. Piperacillin-Tazobactam

Question 2:

You are treating 34-year-old female admitted for pyelonephritis which has been complicated by bacteremia. Which of the following organisms would ceftriaxone most likely cover?

A. *Enterococcus faecalis*

**B. *Escherichia coli***

C. *Pseudomonas aeruginosa*

Question 3:

You escalate CeCe's antibiotics from Amoxicillin to Ceftriaxone. Which potential causative organism are you most likely still not covering?

A. *Haemophilus influenzae*

B. *Methicillin susceptible Staphylococcus aureus*

C. *Moraxella catarrhalis*

**D. *Mycoplasma pneumoniae***

E. *Streptococcus pneumoniae*

**Video 4 – Vancomycin and Azithromycin:**

Question 1:

Would azithromycin be appropriate coverage for suspected community acquired pneumonia in a previously healthy 32-year-old?

**A. Yes**

B. No

Question 2:

You go ahead and use azithromycin for your previously healthy 32-year-old patient with community acquired pneumonia (CAP). Which of the following possible causative organisms for CAP would Azithromycin most likely NOT offer adequate coverage?

A. *Chlamydophila pneumoniae*

B. *Haemophilus influenzae*

C. *Mycoplasma pneumoniae*

**D. *Staphylococcus aureus***

E. *Streptococcus pneumoniae*

Question 3:

We know that in CeCe's case, the main reason we are adding vancomycin to ceftriaxone is that ceftriaxone does not cover methicillin resistant *Staphylococcus aureus*. What other organism (unlikely to cause community acquired pneumonia) does vancomycin most likely cover that ceftriaxone does not?

A. *Enterobacter cloacae*

**B. *Enterococcus faecalis***

C. *Escherichia coli*

D. *Proteus mirabilis*

E. *Pseudomonas aeruginosa*

Question 4:

You are treating a patient who has a methicillin resistant *Staphylococcus aureus* pneumonia with vancomycin. You check the trough before the fourth dose and note that the level is supratherapuetic. Which of following laboratory studies is most important to check in the coming days?

**A. Basic metabolic panel**

B. Coagulation studies

C. Complete blood count

D. Hepatic function panel

E. Sedimentation Rate

**Video 5 - Piperacillin-Tazobactam and Ampicillin-Sulbactam:**

Question 1:

You are taking care of a patient with septic shock from a urinary source on ceftriaxone but are thinking about escalating to piperacillin-tazobactam. What is piperacillin-tazobactam most likely covering that ceftriaxone is not?

A. *Escherichia coli*

B. *Klebsiella [Enterobacter] aerogenes*

C. *Klebsiella pneumoniae*

D. *Methicillin resistant Staphylococcus aureus*

**E. *Pseudomonas aeruginosa***

Question 2:
You are treating an inpatient for a hospital acquired pneumonia with piperacillin-tazobactam. Your attending wants to change antibiotics to ampicillin-sulbactam but you are concerned that you are likely losing coverage for which of the following organisms?

A. *Acinetobacter baumannii*

B. *Bacteroides fragilis*

C. *Enterobacter cloacae*

D. *Enterococcus faecalis*

**E. *Pseudomonas aeruginosa***

Question 3:

You are treating an older gentleman in septic shock from suspected GI source with piperacillin-tazobactam. If you deescalate the patient from piperacillin-tazobactam to ceftriaxone, which of the following organisms will you most likely no longer be covering?

**A. *Bacteroides fragilis***

B. *Escherichia coli*

C. *Klebsiella aerogenes*

D. *Klebsiella pneumoniae*

E. *Proteus mirabilis*

Question 4:
BONUS QUESTION: When switching from ampicillin-sulbactam to amoxicillin-clavulanate you are losing coverage for which organism?

**A. *Acinetobacter baumannii***

B. *Bacteroides fragilis*

C. *Enterobacter cloacae*

D. *Enterococcus faecalis*

E. *Pseudomonas aeruginosa*

**Video 6 - Cefepime:**

Question 1:

You are treating a patient with septic shock and are concerned about Gram negative rod resistance. You change antibiotics from piperacillin-tazobactam to cefepime but are most likely to lose coverage for which of the following organisms?

**A. *Bacteroides fragilis***

B. *Enterobacter cloacae*

C. *Klebsiella aerogenes*

D. *Proteus mirabilis*

E. *Pseudomonas aeruginosa*

Question 2:

Species of which of the following are NOT likely to have an AmpC resistance pattern?

A. *Acinetobacter*

B. *Citrobacter*

**C. *Enterococcus***

D. *Indole positive Proteus*

E. *Serratia*

Question 3:
BONUS QUESTION: What is another cephalosporin antibiotic (technically a third generation) that also covers *Pseudomonas*?

A. Cefazolin

B. Ceftaroline

**C. Ceftazidime**

D. Ceftriaxone

**Video 7 - Aminoglycosides:**

Question 1:

Which of the following is NOT an aminoglycoside?

A. Amikacin

**B. Clindamycin**

C. Gentamicin

D. Streptomycin

E. Tobramycin

Question 2:

Often, aminoglycosides are used in combination with beta-lactams. Which of the following infections would it be appropriate to treat with an aminoglycoside alone?

A. *Enterococcus* bacteremia

B. *Enterococcus* urinary tract infection

C. *Klebsiella* bacteremia

D. *Klebsiella* meningitis

**E. *Klebsiella* urinary tract infection**

Question 3:
You are treating a patient with septic shock from suspected GI vs urinary source in the intensive care unit with vancomycin, cefepime and gentamicin. What organism are you most likely still not covering?

**A. *Bacteroides fragilis***

B. *Enterococcus faecalis*

C. *Enterobacter cloacae*

D. *Escherichia coli*

E. *Pseudomonas aeruginosa*

**Video 8 - Carbapenems:**

Question 1:

Compared with cefepime, meropenem may have broader Gram negative coverage based on exposure history. What else does meropenem most likely cover that cefepime does not?

**A. *Bacteroides fragilis***

B. *Clostridium difficile*

C. Methicillin resistant *Staphylococcus aureus*

D. *Mycoplasma pneumoniae*

E. *Vancomycin resistant Enterococcus faecalis*

Question 2:
You are treating a patient for an intra-abdominal infection complicated by bacteremia and the culture results as an Extended-spectrum beta-lactamase producing *E Coli*. Which of the following antibiotics would be most likely to be effective?

A. Cefazolin

B. Ceftaroline

C. Ceftriaxone

**D. Ertapenem**

E. Piperacillin-Tazobactam
