# Supplementary material for: The Stairway to Antibiotic Heaven: A Scaffolded Video Series on Empiric Antibiotic Selection for Fourth-Year Medical Students
Source: MedEdPORTAL. 2020 Nov 30;16:11036. doi: 10.15766/mep_2374-8265.11036 (PMC7703485; doi:10.15766/mep_2374-8265.11036)
Supplement: Supplementary file 1 — Video 1-Introduction.mp4Video 2-Amoxicillin.mp4Video 3-Ceftriaxone.mp4Video 4-Vancomycin and Azithromycin.mp4Video 5-Piperacillin-Tazobactam and Ampicillin-Sulbactam.mp4Video 6-Cefepime.mp4Video 7-Aminoglycosides.mp4Video 8-Carbapenems.mp4Embedded Questions.docxPre- and Posttest Question Bank.docxPostvideo Survey.docx [file mep_2374-8265.11036-s001.zip › J. Pre- and Posttest Question Bank.docx]

Pre-Test and Post-Test Question Bank

Start of Block: Introduction

A 55-year-old man presents to your clinic with a non-purulent cellulitis and you are concerned about Gram positive cocci. Which of the following organisms is a likely pathogen?

A. *Enterobacter cloacae*

B. *Haemophilus influenzae*

C. *Moraxella catarrhalis*

D. *Neisseria meningitidis*

***E. Streptococcus pyogenes***

A 97-year-old female is hospitalized with pyelonephritis and bacteremia. Gram stain of her blood cultures reveals Gram negative rods.  Which of the following is the most likely pathogen?

*A. Clostridium difficile*

*B. Enterococcus faecalis*

***C. Klebsiella pneumoniae***

*D. Legionella pneumophila*

*E. Listeria monocytogenes*

Beta-lactam antibiotics lack activity against so-called “atypical” organisms because “atypical” organisms…

Choose the correct answer.

**A. Do not have susceptible cell walls**

B. Have efflux pumps

C. Have a thicker cell membrane

D. Mutate their penicillin-binding proteins

E. Produce beta-lactamases

You are taking care of a 23-year-old woman in your outpatient clinic who is at twelve weeks’ gestation and has recently tested positive for syphilis. What is the most appropriate antibiotic to treat her with?

A. Ceftriaxone

B. Doxycycline

C. Levofloxacin

D. Meropenem

**E. Penicillin**

End of Block: Introduction

Start of Block: Amoxicillin

If you are treating your outpatient pediatric patient who has a productive cough and fever with amoxicillin you are most likely concerned about which organism?

A. *Moraxella catarrhalis*

B. *Mycoplasma pneumoniae*

C. *Staphylococcus aureus*

**D. *Streptococcus pneumoniae***

E. *Streptococcus pyogenes*

What is the most common mechanism in *Staphylococcus aureus* leading to resistance to amoxicillin?

A. Lack of cell wall

B. Mutation of their penicillin binding proteins

**C. Production of beta-lactamase**

D. Production of efflux pumps

E. Thickening of cell membrane

Which of the following organisms will amoxicillin most likely cover?

A. *Bacteroides fragilis*

**B. *Haemophilus influenzae***

C. *Legionella pneumophila*

D. *Moraxella catarrhalis*

E. *Mycoplasma pneumoniae*

You are treating an asymptomatic pregnant woman with Group B *Streptococcus* (greater than 100,000 CFU) growing in her urine. What is the most appropriate antibiotic to prescribe?

**A. Amoxicillin**

B. Cefepime

C. Ceftriaxone

D. Gentamicin

E. Levofloxacin

End of Block: Amoxicillin

Start of Block: Ceftriaxone

You are admitting a thirty-year-old woman with fever and altered mental status. Lumbar puncture is performed with an elevated white blood cell count (90% neutrophils), and Gram stain revealing Gram negative diplococci. Which of the following would be the most appropriate antibiotic choice as part of her treatment regimen?

A. Amoxicillin

B. Azithromycin

C. Cefazolin

**D. Ceftriaxone**

E. Piperacillin-tazobactam

A sixty-eight-year-old man presents to the hospital with dysuria and urinary hesitancy. Digital rectal exam reveals a tender prostate. The patient is started on ceftriaxone in the emergency department and is admitted to a medicine service. Urinary cultures subsequently grow *Enterococcus faecalis*. What is the most appropriate course of action?

A. Add an aminoglycoside

**B. Deescalate antibiotics to ampicillin**

C. Escalate antibiotics to cefepime

D. Maintain patient on ceftriaxone

E. Switch antibiotics to levofloxacin

Which of the following is a reason why we consider ceftriaxone a “step-up” from amoxicillin for community acquired pneumonia?

A. Amoxicillin does not cover *Streptococcus pneumoniae*.

B. Ceftriaxone is likely to cover methicillin resistant *Staphylococcus aureus* but amoxicillin is not.

C. Ceftriaxone is likely to cover *Pseudomonas* but amoxicillin is not.

**D. Ceftriaxone offers a broader spectrum of coverage compared with amoxicillin.**

E. Enteric gram negative rods are more likely to be sensitive to ceftriaxone.

You are treating a clinic patient who is positive for influenza A. You are treating her supportively and she is improving but suddenly she started developing high fevers, worsening cough and difficulty breathing. You are most concerned about which organism?

A. *Haemophilus influenzae*

B. *Legionella pneumophila*

C. *Mycoplasma pneumoniae*

**D. *Staphylococcus aureus***

E. *Streptococcus pyogenes*

End of Block: Ceftriaxone

Start of Block: Vancomycin and Azithromycin

You are taking care of a 76-year-old man hospitalized for community acquired pneumonia. She has already been started on ceftriaxone, but you add azithromycin because you are concerned about which organism?

A. *Haemophilus influenzae*

B. *Moraxella catarrhalis*

**C. *Mycoplasma pneumoniae***

D. *Pseudomonas aeruginosa*

E. *Streptococcus pneumoniae*

A previously healthy thirty-year-old female presents to your office with fever, productive cough and decreased breath sounds in the left lower lung field. She does not have any indications for inpatient admission. What would be the most appropriate antibiotic choice?

A. Ampicillin

**B. Azithromycin**

C. Ceftriaxone

D. Metronidazole

E. Piperacillin-tazobactam

Which organism does vancomycin most likely cover that ceftriaxone does not?

A. *Bacteroides fragilis*

B. Extended spectrum beta-lactamase producing *Klebsiella pneumoniae*

**C. Methicillin resistant *Staphylococcus aureus***

D. *Pseudomonas aeruginosa*

E. *Streptococcus pneumoniae*

You are taking care of a seventy-four-year-old man with pneumonia on the general medicine wards. The patient was started on vancomycin and ceftriaxone by the emergency department. What test can you send to feel more confident that you can discontinue the vancomycin?

A. Blood cultures

**B. Methicillin resistant *Staphylococcus aureus* nasal swab**

C. Multi drug resistant organism rectal swab

D. *Streptococcus pneumoniae* urine antigen

E. Respiratory Pathogen Panel

End of Block: Vancomycin and Azithromycin

Start of Block: Piperacillin-tazobactam and Ampicillin-sulbactam

You are taking care of a 78-year-old woman with multiple co-morbidities who has been in the hospital for one week with a heart failure exacerbation. She has a Foley catheter in place. On hospital day eight she has a fever and is started on ceftriaxone. The hospital laboratory calls you and reports that her blood cultures are growing *Pseudomonas aeruginosa*. What is the most appropriate course of action (in addition to discontinuing the Foley catheter)?

A. Add vancomycin to ceftriaxone

B. Change antibiotics to ampicillin-sulbactam

C. Change antibiotics to ceftaroline

**D. Change antibiotics to piperacillin-tazobactam**

E. Maintain the patient on ceftriaxone

54-year-old woman presents to the hospital with fever, right upper quadrant pain and jaundice. She is found to be in septic shock and is admitted to the intensive care unit. Since you are concerned about cholangitis, what would be the most appropriate empiric antibiotic choice?

A. Ampicillin

B. Azithromycin

C. Ceftriaxone

**D. Piperacillin-tazobactam**

E. Vancomycin

You are in the surgical intensive care unit taking care of a patient status post Whipple procedure that has been complicated by intra-abdominal collections growing *Bacteroides fragilis*. Which of the following antibiotics is most likely to provide adequate coverage?

**A. Ampicillin-sulbactam**

B. Azithromycin

C. Aztreonam

D. Cefepime

E. Ceftriaxone

Which organism may piperacillin-tazobactam cover that ampillicin-sulbactam does not?

A. *Bacteroides fragilis*

B. *Enterococcus faecalis*

C. *Klebsiella pneumoniae*

**D. *Pseudomonas aeruginosa***

E. Methicillin susceptible *Staphylococcus aureus*

End of Block: Piperacillin-tazobactam and Ampicillin-sulbactam

Start of Block: Cefepime

Certain species of which genera are most likely to have an AmpC beta-lactamase resistance pattern?

A. *Bacteroides*

B. *Enterococcus*

C. *Neisseria*

D. *Serratia*

E. *Staphylococcus*

You are taking care of a 64-year-old woman with septic shock who has *Citrobacter freundii* growing in her urine and blood cultures. The patient is currently on vancomycin and ceftriaxone. Looking at the sensitivities of the organism, you identify an AmpC beta-lactamase pattern. What is the most appropriate action?

A. Change ceftriaxone to ampicillin

**B. Change ceftriaxone to cefepime**

C. Change ceftriaxone to polymyxin

D. Maintain the patient on vancomycin and ceftriaxone

E. Maintain the patient on vancomycin but discontinue ceftriaxone

When you change antibiotics from piperacillin-tazobactam to cefepime, you are most likely losing coverage for which of the following organisms?

**A. *Enterococcus faecalis***

B. *Enterobacter cloacae*

C. *Mycoplasma pneumoniae*

D. *Pseudomonas aeruginosa*

E. *Streptococcus pyogenes*

You are taking care of a patient in septic shock and you are concerned about a GI source. The patient is currently on vancomycin and cefepime. Which antibiotic would be most appropriate to add?

A. Ampicillin

B. Azithromycin

C. Ciprofloxacin

**D. Metronidazole**

E. Trimethoprim-sulfamethoxazole

End of Block: Cefepime

Start of Block: Aminoglycosides

What is the main mechanism of action of aminoglycosides?

A. Blocking RNA synthesis

B. Disruption of cell wall formation

**C. Inhibiting protein synthesis**

D. Interfering with DNA replication

E. Interfering with folic acid metabolism

Which of the following *Escherichia coli* infections would potentially be appropriate to treat with aminoglycoside monotherapy?

A. Bacteremia

B. Intra-abdominal abscess

C. Meningitis

D. Osteomyelitis

**E. Urinary tract infection**

Aminoglycoside monotherapy most likely would cover which of the following organisms?

A. *Enterococcus faecalis*

B. *Legionella pneumophila*

**C. *Proteus mirabilis***

D. *Staphylococcus aureus*

E. *Streptococcus pneumoniae*

You are taking care of a patient in intensive care unit with septic shock secondary to a urinary source. Blood cultures are positive for *Escherichia coli*, but sensitivities have not returned. The patient is currently on piperacillin-tazobactam and remains on multiple pressor medications. Which of the following is the most appropriate antibiotic to add until sensitivities return?

A. Azithromycin

B. Clindamycin

**C. Gentamicin**

D. Metronidazole

E. Vancomycin

End of Block: Aminoglycosides

Start of Block: Carbapenems

You are taking care of a 67-year-old female in the intensive care unit who has *Escherichia coli* growing in her blood. You see that the organism appears sensitive to cefoxitin but resistant to ceftriaxone. The patient is currently on ceftriaxone. What is the most appropriate course of action?

A. Add azithromycin to ceftriaxone

B. Add vancomycin to ceftriaxone

C. Change ceftriaxone to cefoxitin

**D. Escalate from ceftriaxone to meropenem**

E. Escalate from ceftriaxone to polymyxin

When you deescalate antibiotics from meropenem to ertapenem, you lose coverage of which organism?

A. *Enterobacter cloacae*

B. *Escherichia coli*

C. *Proteus mirabilis*

**D. *Pseudomonas aeruginosa***

E. *Streptococcus pneumoniae*

Compared with cefepime, you most likely gain coverage of which organism when you escalate to meropenem?

**A. *Bacteroides fragilis***

B. *Enterobacter cloacae*

C. *Methicillin resistant Staphylococcus aureus*

D. *Mycoplasma pneumoniae*

E. *Pseudomonas aeruginosa*

You are working in the intensive care unit when the hospital laboratory calls you and tells you one of your patients has a Klebsiella pneumoniae carbapenemase (KPC) producing *Klebisella pneumoniae* (KPC - KP). Which of the following is most likely active against a KPC organism?

A. Cefepime

B. Ertapenem

C. Meropenem

D. Piperacillin-Tazobactam

**E. Polymyxin**

End of Block: Carbapenems
