# Supplementary material for: The Stairway to Antibiotic Heaven: A Scaffolded Video Series on Empiric Antibiotic Selection for Fourth-Year Medical Students
Source: MedEdPORTAL. 2020 Nov 30;16:11036. doi: 10.15766/mep_2374-8265.11036 (PMC7703485; doi:10.15766/mep_2374-8265.11036)
Supplement: Supplementary file 1 — Video 1-Introduction.mp4Video 2-Amoxicillin.mp4Video 3-Ceftriaxone.mp4Video 4-Vancomycin and Azithromycin.mp4Video 5-Piperacillin-Tazobactam and Ampicillin-Sulbactam.mp4Video 6-Cefepime.mp4Video 7-Aminoglycosides.mp4Video 8-Carbapenems.mp4Embedded Questions.docxPre- and Posttest Question Bank.docxPostvideo Survey.docx [file mep_2374-8265.11036-s001.zip › K. Postvideo Survey.docx]

Empiric Antibiotics

Start of Block: "Empiric Antibiotics" Post Video Survey

Q1 Which "Empiric Antibiotics" video are you rating?

- Intro (Video 1 – Introduction)
- Beta Lactams 1 (Video 2 - Amoxicillin)
- Beta Lactams 2 (Video 3 - Ceftriaxone)
- Covering the Bases (Video 4 - Vancomycin and Azithromycin)
- Beta Lactams 3 (Video 5 – Piperacillin-Tazobactam and Ampicillin-Sulbactam)
- Beta Lactams 4 (Video 6 - Cefepime)
- Double Trouble (Video 7 - Aminoglycosides)
- Beta Lactams 5 (Video 8 - Carbapenems)

Q2

|  | Strongly Disagree | Disagree | Neutral | Agree | Strongly Agree |
| --- | --- | --- | --- | --- | --- |
| This video module was an effective way to learn about antibiotic coverage |  |  |  |  |  |
| The length of this video module was appropriate |  |  |  |  |  |
| The video module had an appropriate level of difficulty |  |  |  |  |  |
| I enjoyed this video module |  |  |  |  |  |
| The questions within the video module were a helpful way to reinforce the material |  |  |  |  |  |

Q3

|  | Strongly Disagree | Disagree | Neutral | Agree | Strongly Agree |
| --- | --- | --- | --- | --- | --- |
| After watching this module, I am more likely to remember the spectrum of activity of the presented antibiotics |  |  |  |  |  |
| After watching this module, I better understand why the presented antibiotics are selected for empiric coverage |  |  |  |  |  |
| After watching this module, I feel more comfortable using the presented antibiotics in the appropriate clinical setting |  |  |  |  |  |

Q4 I am a...

- Pre-Clinical CUMC medical student
- MCY or above CUMC medical student
- Internal Medicine PGY-1
- Internal Medicine PGY-2/3
- Pediatrics PGY-1
- Pediatrics PGY-2/3
- Fellow
- Attending
- Other

Please share any comments or feedback you have about this video module.
Feel free to mention potential errors or typos--these are still in development.

________________________________________________________________

________________________________________________________________

________________________________________________________________

________________________________________________________________

________________________________________________________________

End of Block: "Empiric Antibiotics" Post Video Survey
